# Influence of *Lactobacillus plantarum* inoculation on the silage quality of intercropped *Lablab purpureus* and sweet sorghum grown in saline-alkaline region

**DOI:** 10.3389/fmicb.2022.1059551

**Published:** 2022-12-02

**Authors:** Huangkeyi Li, Tianwei Wang, Muhammad Tahir, Jiaqi Zhang, Jiahao Sun, Tianqi Xia, Fuqing Huang, Yayong Liu, Zhiquan Liu, Jin Zhong

**Affiliations:** ^1^School of Life Sciences, Yunnan University, Kunming, China; ^2^State Key Laboratory of Microbial Resources, Institute of Microbiology, Chinese Academy of Sciences, Beijing, China; ^3^School of Life Sciences, University of Chinese Academy of Sciences, Beijing, China; ^4^State Key Laboratory of Vegetation and Environmental Change, Institute of Botany, Chinese Academy of Sciences, Beijing, China

**Keywords:** *Lablab purpureus*, silage quality, *Lactobacillus plantarum*, intercropped, microbial community

## Abstract

Ensiling legume with cereal is an effective method to ensure the energy rich-feed, but no information is available on the microbial fermentation mechanism of intercropped *Lablab purpureus* (Lablab) and sweet sorghum in the saline-alkaline region. Therefore, the present study investigated the silage quality and microbial community of intercropped Lablab and sweet sorghum silages grown in the saline-alkaline region with or without inoculation of *Lactobacillus plantarum* (LP). The experimental treatments were prepared according to the Lablab and sweet sorghum planting patterns: Lablab and sweet sorghum sowing seed ratios were 1:1 (L), 5:1 (M), and 9:1 (H). After harvesting, each mixture was treated with LP or sterilized water (CK), followed by 60 days of fermentation. Results showed that both LP inoculation and intercropping significantly raised the lactic acid (LA) content and decreased the pH value, acetic acid (AA), and ammonia-N in intercropped silages. The LP addition and intercropping also improved the relative feed value by reducing structural carbohydrates. Moreover, LP silages had a greater relative abundance of *Lactobacillus* than CK silages, and its relative abundance increased with an increased seed-sowing ratio of Lablab in intercropping. LP was the prevalent species in LP silages compared to CK silages, and its relative abundance also increased with an increased seed-sowing ratio of Lablab in intercropping. The genus *Lactobacillus* was negatively correlated with ammonia-N (R = −0.6, *p* = 0.02) and AA (R = −0.7, *p* < 0.01) and positively correlated with LA (R = 0.7, *p* < 0.01) and crude protein (R = 0.6, *p* = 0.04). Overall, the intercropped seeding ratios of Lablab and sweet sorghum of ≥ 5:1 with LP inoculation resulted in better fermentation quality and preservation of nutritional components providing theoretical support and guidance for future intercropped protein-rich silage production in the saline-alkaline region.

## Introduction

Ensiling is the most promising forage preservation method which assures ruminants year-round access to high-quality silage (Pahlow et al., 2003; Dunière et al., 2013). The preservation of forages that are rich in protein is also particularly crucial since plant-derived proteins are necessary for livestock products (Xia et al., 2022). However, the development of animal husbandry is generally limited in the saline-alkaline region due to a deficit in protein-rich forage. This shortage of protein-rich forage is generally induced by alkali or saline stress which affects the typical plants' nutrient uptake (Hou et al., 2018). Consequently, cultivating and utilizing protein-rich forages to replace the protein-poor forages in the saline-alkali region is imperative.

The *Lablab purpureus* (Lablab) is an important perennial legume worldwide, characterized by the greater protein content and resistance to salt or alkali stress (Hailin et al., 2017). The Lablab is a dual-purpose legume, which is consumed by humans as a pulse or used as fodder for livestock (Mudryj et al., 2014; Rebello et al., 2014). However, the natural fermentation of Lablab often leads to failure due to its high buffer capacity and low sugar content (Yu, 2015). Sweet sorghum is abundant with a fermentable carbohydrate content of ~20% dry matter (DM) and can tolerate salt or alkali stress (Khota et al., 2017; Chen et al., 2019). Sweet sorghum silage is prevalent due to its low buffering capacity and high content of water-soluble carbohydrates, making it easy to ensile into energy-rich feed (Dong et al., 2020), but its protein content is insufficient. A combination of legume with cereal silage (soybean and corn) has been effective because the high protein content of the soybean increases the nutritional value while the abundant carbohydrates of the corn offer enough substrate for lactic acid bacteria (LAB) to proliferate, ensuring quality fermentation of the intercropped forages (Zeng et al., 2020). Similarly, mixed silages of sweet sorghum with alfalfa, corn, and *Sesbania cannabina* have been proven successful (Zhang et al., 2015; Ni et al., 2018; Xia et al., 2022). However, in these earlier trials, conventional legumes and sweet sorghum were sown and harvested separately and then ensiled at different weight ratios. Intercropped crops can be ensiled directly in accordance with the planting patterns rather than the weight ratio, thereby saving labor, money, and storage space (Zeng et al., 2022). Therefore, it is imperative to understand the microbial fermentation mechanism of intercropped Lablab and sweet sorghum according to their planting system with multiple benefits.

The LAB additives are largely used for quality silage production because of their ability to produce lactic acid (LA), decrease the pH, inhibit protein degradation, and reduce DM loss under anaerobic conditions (Filya et al., 2006; Oliveira et al., 2017). Generally, homofermentative LAB strains are the major contributor to LA-type fermentation due to their greater efficiency of sugar utilization. It has been established that the rapid growth of *Lactobacillus (L.) plantarum (LP)* during the initial phase of fermentation is critical for the later fermentation and final silage quality (Yang et al., 2019). Numerous studies showed that the addition of LP ensures successful fermentation by restricting the undesired microorganisms (EFSA Panel on Additives Products or Substances used in Animal Feed (FEEDAP) et al., 2017; Oliveira et al., 2017; Yang et al., 2020; Zhang et al., 2022). However, little is known about the influence of LP on the composition of intercropped Lablab and sweet sorghum silage, which is essential for further regulation of fermentation.

Consequently, the present study investigated the silage quality and microbial community of intercropped Lablab and sweet sorghum silages grown in the saline-alkaline region with or without LP inoculation. In the present study, we hypothesized that intercropping and LAB addition had beneficial effects on the fermentation quality and microbial community of intercropped Lablab and sweet sorghum silage. The results of this study may provide theoretical support and guidance for future protein-rich silage production in the saline-alkaline region with the help of intercropping and the addition of suitable LAB inoculant.

## Materials and methods

### Materials and silage preparation

The Lablab and sweet sorghum fresh materials were collected from the experimental field of Yellow River Delta Modern Agricultural Technology Innovation Center located in Guangrao County, Dongying city, Shandong province, China (118°90' E, 37°67' N) on 20 October 2020. The study area has the following soil characteristics: 9.07 pH, 21.4 g kg^−1^ of organic matter, 1.1 g kg^−1^ total salt, 62.6 mg kg^−1^ of hydrolyzed nitrogen, 1.08 × 10^3^ mg kg^−1^ of total nitrogen, 15 mg kg^−1^ of available phosphorous, and 311 mg kg^−1^ of available potassium. The seeds of Lablab and sweet sorghum were mixed at a weight ratio of 1:1 (L), 5:1 (M), and 9:1 (H) before sowing. A self-selected LP WQ-01 (CGMCC No. 13318, LP) strain screened from high-quality sweet sorghum silage was used as a silage additive.

The treatments were referred to the field cropping system of Lablab and sweet sorghum such as L, M, and H. The intercropped Lablab (productive stage) and sweet sorghum (milk-ripe stage) were harvested together and chopped into a particle size of 2 to 4 cm by a crop chopper (ZS-2, Zhongsheng agricultural machinery company, Tangshan, China). The selected LP strain was incubated on Man, Rogosa, Sharpe (MRS) liquid medium (Solaibao Technology Co., LTD., Beijing, China) at 37°C for 48 h under anaerobic conditions. The cultured LP strain was homogeneously sprayed on the mixtures at 5 × 10^6^ CFU/g fresh matter (FM). An equal amount of sterilized water was sprayed on the mixtures for the control group (CK). The 400 g of samples were packed into the polyethylene bags and tightly vacuum-sealed with a Vacuum machine (DZ-AS, 2,500 KW, ANSEN, Fujian, China). A total of 18 bags (2 groups × 3 ratios × 3 replicates) were prepared and kept at a temperature between 20 and 25°C. The bags were unpacked after 60 days (d) of fermentation to analyze the chemical composition, fermentation quality, and microbial community of intercropped silages.

### Fermentation quality and chemical composition analysis

The fermentation quality parameters analysis was performed according to the previous study (Wang et al., 2020). In brief, 10 g of silage samples and 90 ml of sterilized water were blended for 20 min and passed through a filter (0.22 μm). The extracting liquid was used to examine the pH, organic acids including LA, acetic acid (AA), propionic acid (PA), butyric acid (BA), and ammonia-N. A pH value was determined by using the glass electrode pH meter (pH 213; HANNA; Italy). The organic acids contents were analyzed using the high-performance liquid chromatography (HPLC) (column: ICSep COREGEL-87H; detector: 210 nm UV; mobile stage: 0.005 M of H_2_SO_4_, 0.6 mL/min; temperature: 55°C; 1,200, Agilent, America). The ammonia-N concentration was analyzed *via* the ninhydrin colorimetric and phenol-hypochlorite protocols (Broderick and Kang, 1980).

The pre- and post-ensiling samples were dried at 65°C for 48 h in a forced-air oven until a constant weight to estimate the DM content. The dried silage samples were grounded into a particle diameter of 1.0 mm for nutritional components analysis. The crude protein (CP) was measured by following the method of the Association of Official Analytical Chemists (AOAC and AOMA, 1990). The level of the neutral detergent fiber (NDF), acid detergent fiber (ADF), and acid detergent lignin (ADL) were examined according to the previous work (Van Soest et al., 1991).

### Relative feed value analysis

The relative feed value (RFV) was estimated by digestible dry matter (DDM) and dry matter intake (DMI) according to Sun et al. (2021) using the following formula:


$$\begin{array}{l} DDM\;\left(\%DM\right)\;=\;88.90-0.779\times ADF(\%DM)\\ DMI\left(\%Body\;weight\right)\;=\;120/NDF(\%DM)\\ RFV\;=\;(DDM\times DMI)/1.29 \end{array}$$

### Sequencing and bacterial community analysis

A total of 10 g of samples were blended with sterile PBS solution (40 mL) and then shaken at 120 rpm for 30 min at 4°C. The mixed solution was passed through a filter (0.22 mm, Cat. B-CYD400G1). Then, the filtrate was centrifuged at 12,000 rpm for 10 min at 4°C. After the above pre-treatments, the bacteria precipitate was collected to extract the total genomic DNA using the DNA isolation kit (Yeasen, 18815ES50, Shanghai, China) (Sun et al., 2021).

The V3–V4 regions of the 16S ribosomal RNA (rRNA) gene were amplified by PCR for high-throughput sequencing using the forward primer 338F (ACTCCTACGGGAGGCAGCA) and the reverse primer 806R (GGACTACHVGGGTWTCTAAT) according to the previous report (Su et al., 2019). To ensure the accuracy of PCR, each sample was set up in three groups for PCR reaction. The mixture of three PCR products was sequenced by using paired terminal read (2 × 300 bp) with the Illumina MiSeq platform (Shanghai Majorbio Biopharm Technology Co. Ltd.) based on standard protocols. The sequencing reads were managed in accordance with the previous report (Ni et al., 2018). To obtain high-quality sequencing, their barcode and primers were discharged, and then Mothur (v.1.34.4) was used to discharge the sequences <200 bp whose maxhomop value is >10. The remaining sequences were checked for chimeras in the *de novo* mode by USEARCH 8.0. After the filtering process, the clean reads remained for downstream analysis. The operational taxonomic units (OTUs) at a similarity level of 97% were clustered using QIIME (v1.8.0). The OTUs file was used to calculate rarefaction [R (v.22)] and alpha diversity [Mothur (v1.34.4)]. The weighted UniFrac distance matrix was employed to calculate principal coordinates analysis (PCoA) which was performed at a 3% dissimilarity level. The raw tags were quality-filtered and merged using Trimmomatic (Version 3.29) and FLASH (Version 1.2.11). ACE, Simpson, Chao, Shannon, and Good's coverage were all calculated to assess alpha diversity (Sun et al., 2021). All the sequences in the current study were deposited to the sequence read archive (SRA) of the NCBI database under the project accession number PRJNA885981.

### Statistical analysis

The statistics were performed using the SAS (version 9.0, 2002; SAS Institute, Cary, NC, USA). The student *t*-test was applied to compare the means of two groups, while a one-way analysis of variance was conducted for multiple groups with Duncan's multiple range test. The relationships between the bacterial taxonomic profile and silage quality variables were determined by calculating the spearman correlation coefficients and were plotted by using the “pheatmap” libraries in R. The significance was employed at a probability level of 0.05.

## Results

### Chemical characteristics of fresh materials

The chemical composition of the intercropped fresh materials is presented in Table 1. The CP, NDF, and ADF contents were significantly (*p* < 0.05) influenced by intercropping. The CP content increased, while NDF and ADF contents decreased with the increased sowing ratio of Lablab in intercropping. The H intercropped ratio had a greater CP content (8.77% DM), while less NDF (48.77% DM) and ADF (31.78% DM) contents than other treatments. However, the ADL content was not influenced by the intercropped ratio.

**Table 1:** Chemical composition of intercropped fresh materials before ensiling.

**Sample**	**CP**	**NDF**	**ADF**	**ADL**
	**(% DM)**	**(% DM)**	**(% DM)**	**(% DM)**
L	7.00^b^	52.03^a^	33.78^a^	4.255^a^
M	6.77^b^	49.28^ab^	32.96^ab^	4.47^a^
H	8.77^a^	48.77^c^	31.78^c^	4.58^a^
SEM	0.2757	0.6248	0.3935	0.0804

L, Lablab and sweet sorghum sowing weight ratio of 1:1; M, Lablab and sweet sorghum sowing weight ratio of 5:1; H, Lablab and sweet sorghum sowing weight ratio of 9:1; CP, crude protein; NDF, neutral detergent fiber; ADF, acid detergent fiber; ADL, acid detergent lignin; and SEM, standard error mean. Means within the same column with different letters is significantly different (*P* < 0.05).

### Fermentation characteristics of intercropped lablab and sweet sorghum silages with or without LP

**Figure 1:**
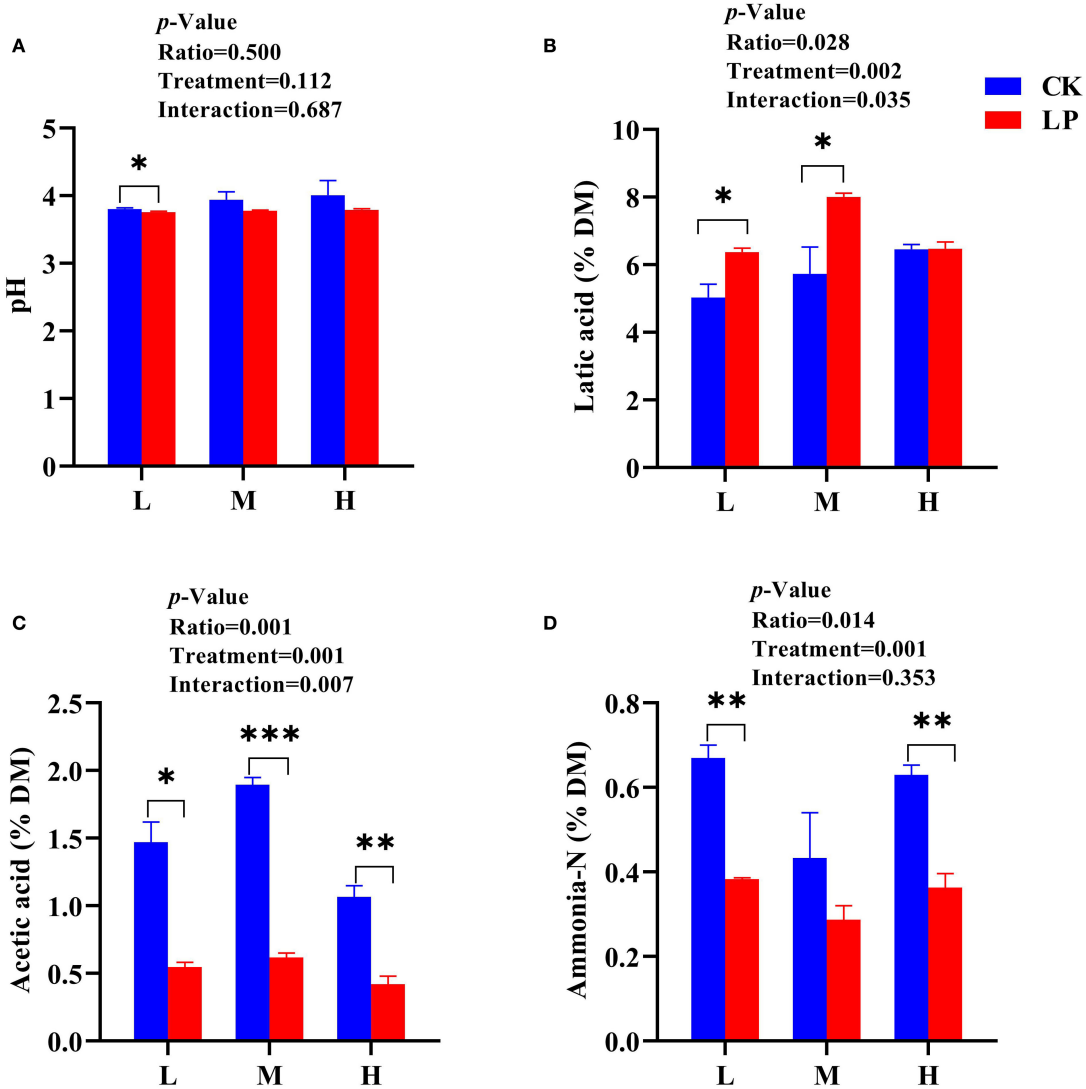
Fermentation profile of Lablab and sweet sorghum intercropped silages with or without LP after fermentation. **(A)** for pH, **(B)** for lactic acid, **(C)** for acetic acid, and **(D)** for ammonia-N. CK, sterile water; LP, *Lactobacillus plantarum*; L, Lablab and sweet sorghum seed-sowing ratio of 1:1; M, Lablab and sweet sorghum seed-sowing ratio of 5:1; and H, Lablab and sweet sorghum seed-sowing ratio of 9:1. “*,” “**,” and “***” represent the significance at 0.05, 0.01, and 0.001, respectively.

The pH values of intercropped silages in the LP group were lower than that of the CK group after 60 d of fermentation (Figure 1A). More specifically, the L intercropped silage had lower pH values both in CK (3.80) and LP (3.75) groups compared to other intercropped silages. All intercropped silages of the LP group except LP-H had greater LA concentrations compared to the CK intercropped silages (Figure 1B). The intercropped silages in the LP group had a lower content of AA compared to the CK group (Figure 1C). The LP-M silage resulted in higher LA content (8% DM), while LP-H showed a lower AA content (0.42% DM) compared to other intercropped silages. The PA and BA were not detected in this study. The content of ammonia-N was substantially lower in the LP group intercropped silages compared to the CK group intercropped silages, and LP-M silage had a lower ammonia-N content compared to other intercropped silages (Figure 1D). Overall, the data presented here highlighted that intercropping ratio of Lablab and LP inoculation significantly influenced the fermentation quality of intercropped Lablab and sweet sorghum silages.

### Chemical composition of intercropped lablab and sweet sorghum silages with or without LP

**Figure 2:**
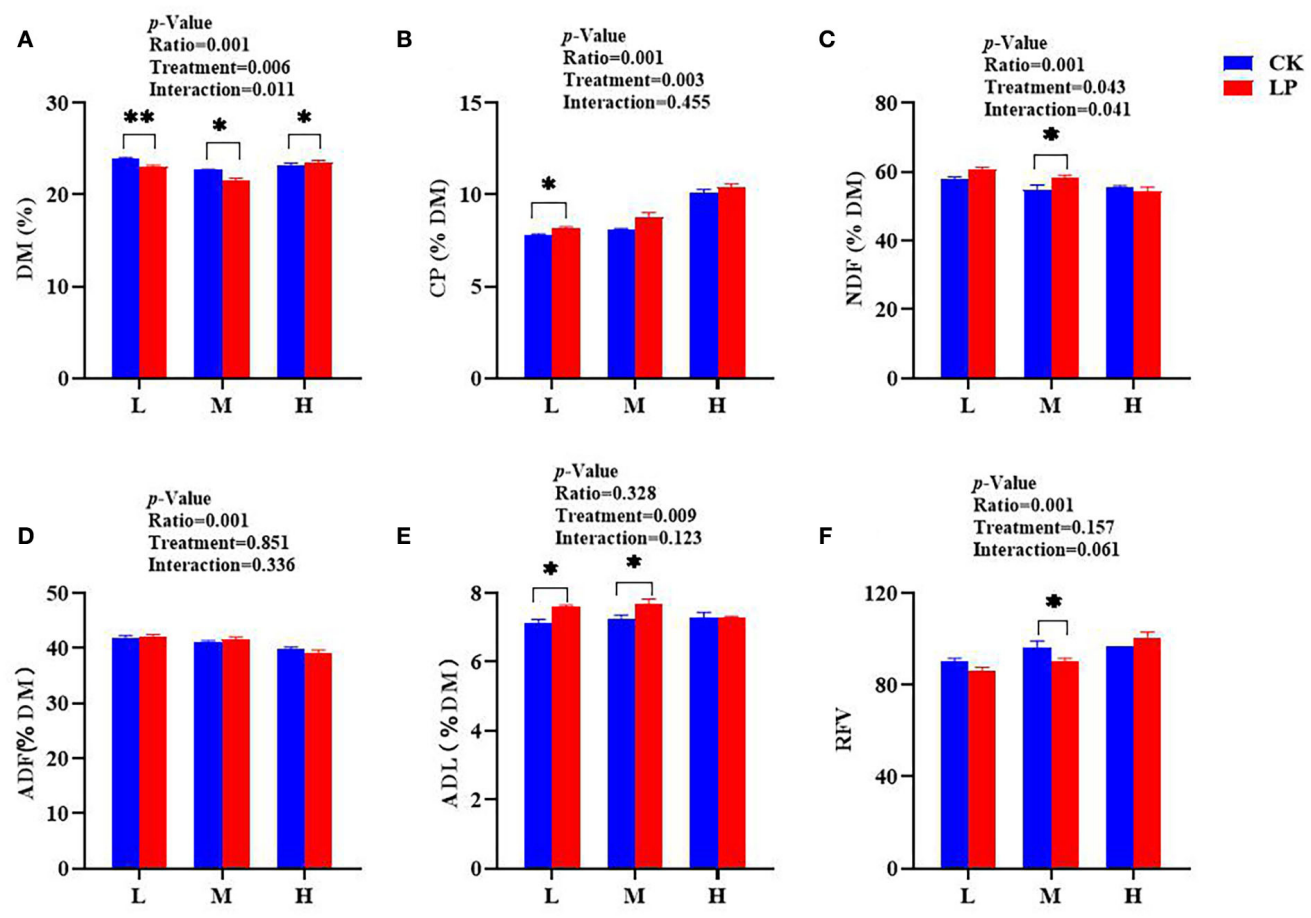
Chemical characteristics of Lablab and sweet sorghum intercropped silages with or without LP after fermentation. **(A)** for DM, **(B)** for CP, **(C)** for NDF, **(D)** for ADF, **(E)** for ADL, and **(F)** for RFV. DM, dry matter; CP, crude protein; NDF, neutral detergent fiber; ADF, acid detergent fiber; ADL, acid detergent lignin. RFV, relative feed value. CK, sterile water; LP, *Lactobacillus plantarum*; L, Lablab and sweet sorghum seed-sowing ratio of 1:1; M, Lablab and sweet sorghum seed-sowing ratio of 5:1; and H, Lablab and sweet sorghum seed-sowing ratio of 9:1. “*” and “**” represent the significance at 0.05 and 0.01, respectively.

The chemical composition of intercropped silages treated with or without LP after 60 d of fermentation is presented in Figure 2. The CK-L silage had a higher DM content compared to other treatments (Figure 2A). The CP concentration in the LP-L group was significantly higher than the CK-L group, and the LP-H silage resulted in greater CP content (10.43% DM) compared to other silages (Figure 2B). The contents of NDF, ADF, and ADL were slightly greater in the LP group except for LP-H compared to the CK group (Figures 2C–E). However, the LP-H silage had significantly lower NDF (54.33% DM) and ADF (39.23% DM) contents compared to other intercropped silages. The RFV increased significantly with an increased ratio of Lablab in both CK and LP groups, and LP-H silage resulted in greater RFV compared to other intercropped silages (Figure 2F). Overall, the data presented here suggested that intercropped silages with a greater ratio of Lablab and LP inoculation resulted in better preservation of chemical components after fermentation.

### Microbial community of intercropped lablab and sweet sorghum silages with or without LP

The alpha diversity of intercropped silages treated with or without LP after 60 d of fermentation is displayed in Supplementary Table 1. The Good's coverage of all groups was over 0.99, which demonstrated that most of the bacterial communities were obtained. The indexes of Shannon and Simpson were lower in the LP group compared with the CK group at each intercropped ratio. According to the PCoA, CK group intercropped silages, except CK-L, were separated from LP group intercropped silages (Supplementary Figure 1). These results showed that the LP inoculation and the ratio of Lablab might have affected the bacterial community of intercropped silages.

**Figure 3:**
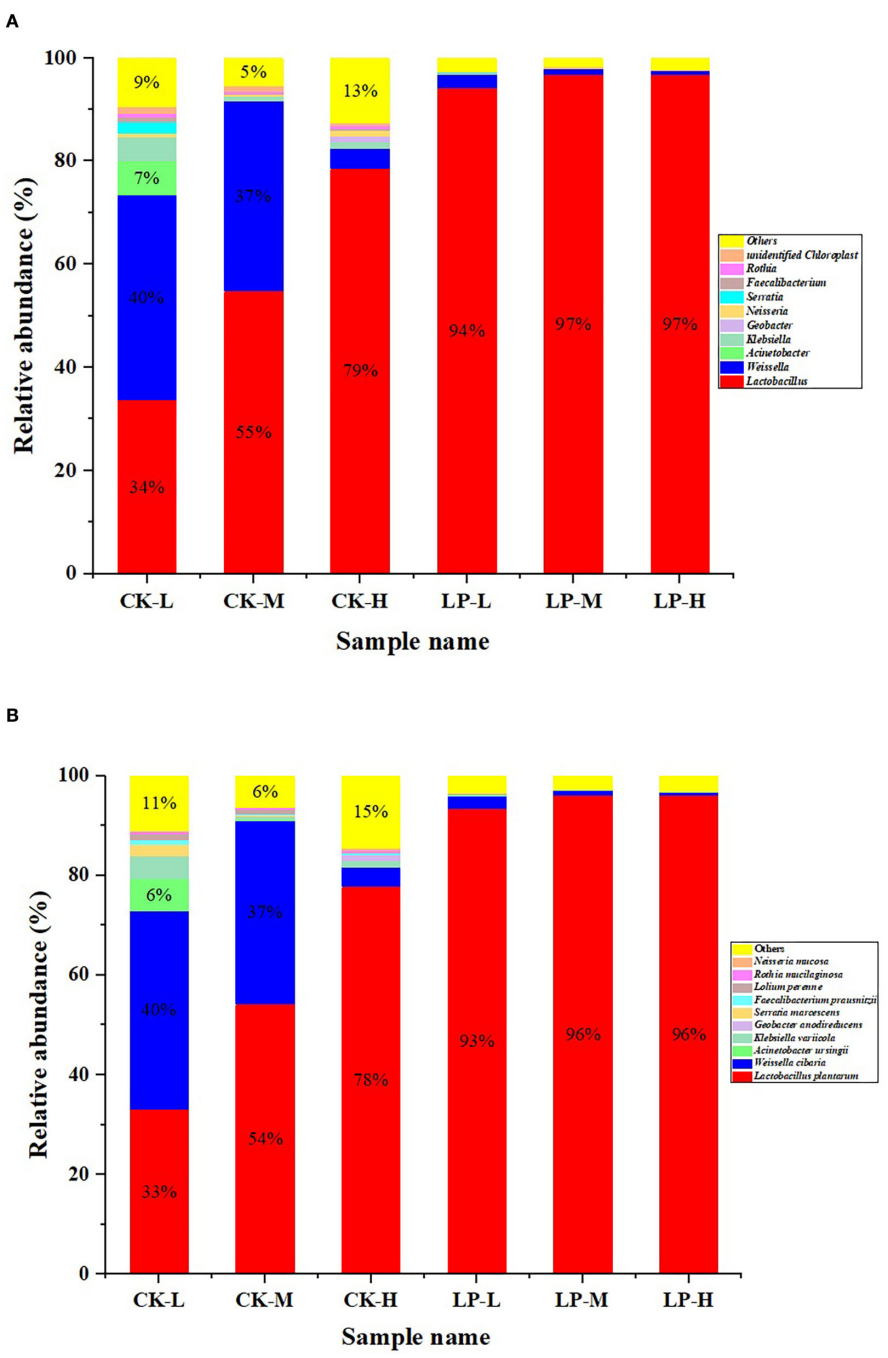
The bacterial community of Lablab and sweet sorghum intercropped silages with or without LP at genus level **(A)** and species level **(B)** after fermentation. CK, sterile water; LP, *Lactobacillus plantarum*; L, Lablab and sweet sorghum seed-sowing ratio of 1:1; M, Lablab and sweet sorghum seed-sowing ratio of 5:1; and H, Lablab and sweet sorghum seed-sowing ratio of 9:1.

The bacterial community of intercropped silages with or without LP inoculation at genus and species levels after 60 d of fermentation is illustrated in Figure 3. The relative abundance of *Lactobacillus* increased with an increase in the Lablab ratio in the CK group (CK-L: 34%, CK-M: 55%, and CK-H: 79%). Contrarily, the relative abundance of *Weissella* decreased with an increase in the Lablab ratio in the CK group (CK-L: 40.17%, CK-M: 37.03%, and CK-H: 4.04%). The similar trends in the relative abundances of *Lactobacillus* (LP-L: 94.76%, LP-M: 97.21%, and LP-H: 97.51%) and *Weissella* (LP-L: 2.59%, LP-M: 1.09%, and LP-H: 0.62%) were also observed in the LP groups. Furthermore, the *Lactobacillus* was the more dominant microbe in the LP group (over 90%) compared to the CK group (Figure 3A). LP (78%) and *Weissella cibaria* (40%) were the dominant species in the CK group intercropped silages but LP was the dominant specie in the LP group intercropped silage (up to 96%). However, the relative abundance of LP increased and the relative abundance of *W. cibaria* decreased gradually with the increase in Lablab ratio in intercropping (Figure 3B).

### Correlation analysis between microbiota and fermentation products

**Figure 4:**
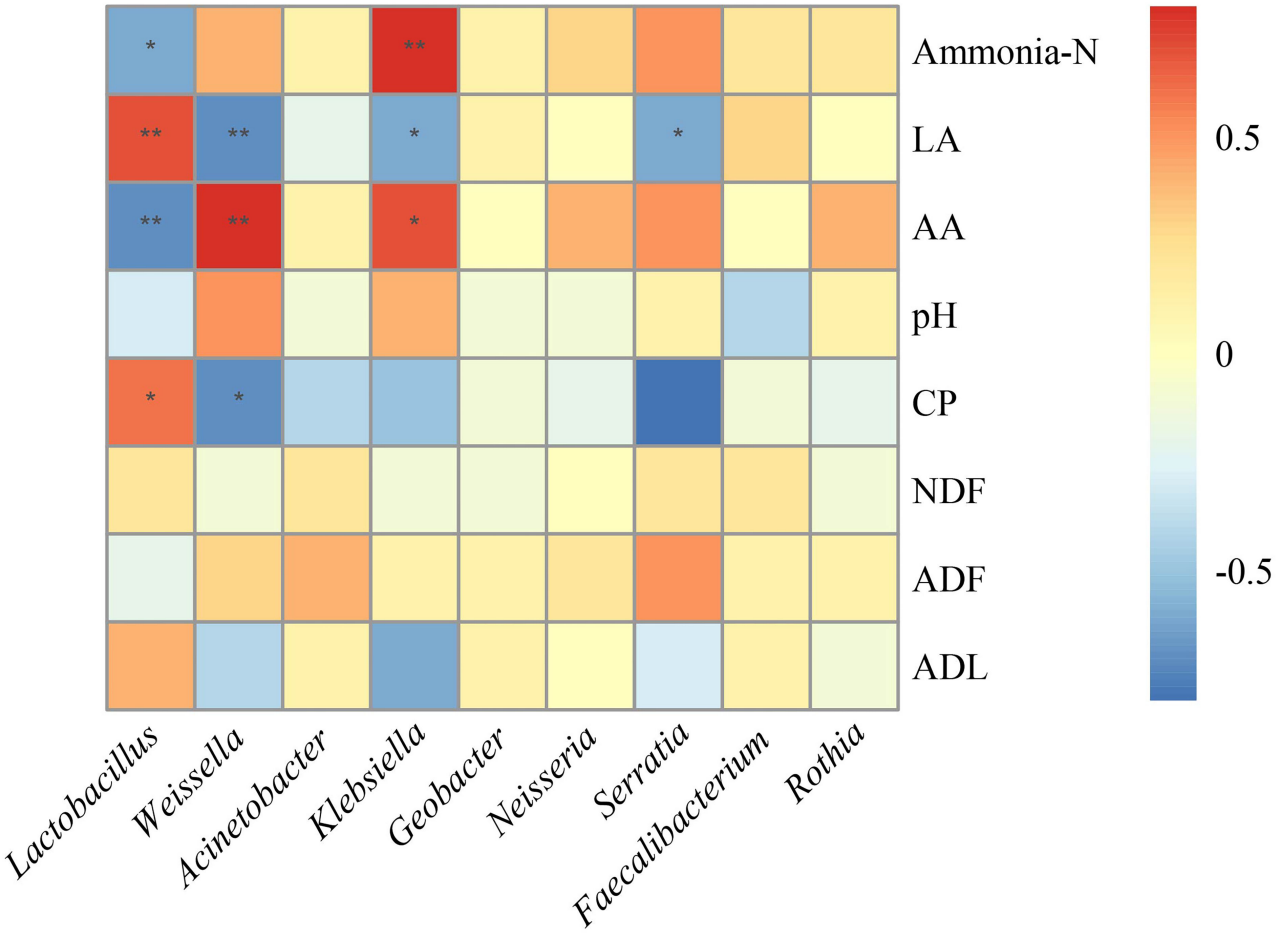
Correlation analysis between bacterial community and fermentation products after fermentation. LA, lactic acid; AA, acetic acid; CP, crude protein; NDF, neutral detergent fiber; ADF, acid detergent fiber; ADL, acid detergent lignin.

To investigate the effects of the microbial community on the fermentation quality, the correlation analysis between microbiota and fermentation products was conducted (Figure 4). The genus *Lactobacillus* was negatively correlated with ammonia-N (R = −0.6, *p* = 0.02) and AA (R = −0.7, *p* < 0.01) and positively correlated with LA (R = 0.7, *p* < 0.01) and CP (R = 0.6, *p* = 0.04). The genus *Weissella* was positively correlated with AA (R = 0.8, *p* < 0.01), while negatively correlated with CP (R = −0.7, *p* = 0.01) and LA (R = −0.7, *p* < 0.01). Moreover, *Klebsiella* was positively correlated with ammonia-N (R = 0.8, *p* < 0.01) and AA (R = 0.7, *p* = 0.03), while negatively correlated with LA (R = −0.6, *p* = 0.03).

## Discussion

The forage silage quality is mainly driven by microbial activity and depends on the substrates present in the fresh materials (Yan et al., 2019). The pH value of silage is a key indicator to assess the fermentation quality of silage (Restelatto et al., 2019), and a pH value below or equal to 4.2 is considered a benchmark for well-fermentation quality (Ni et al., 2018). In the current study, the pH values of both CK and LP groups were lower than 4.2 suggesting that intercropping provided sufficient substrate to LAB to initiate the fermentation (Zhang et al., 2015). Moreover, the pH value of the CK group gradually enhanced with an increased seed-sowing ratio of Lablab in intercropping which might be related to the Lablab's high buffering capacity, which delayed the decrease in pH value. However, the pH value of the LP group was not influenced by the increased seed-sowing ratio of Lablab in intercropping, highlighting that LP inoculation could maintain the pH at a low level in accordance with studies of Arasu et al. (2014) and Ren et al. (2020). The LP-M silage had a greater accumulation of LA after 60 d of fermentation, which may be due to the combined effect of intercropping and LP additive. However, the specific mechanism of interaction on the LA level needs to be further explored. The AA is usually considered the measure of aerobic stability of silage. As expected, the concentrations of AA in the LP group silages were lower in this study, which might be related to the greater relative abundances of LP species in these silages that converts WSC to LA (Yan et al., 2019).

The ammonia-N generally indicates the silage proteolysis during ensiling which is driven by plant enzyme protease and proteolytic *Clostridia* (Su et al., 2019). In the present study, the ammonia-N content was significantly influenced by the intercropping and LP inoculation. The LP silages had a lower ammonia-N content compared to CK silages in agreement with the results of Zhao et al. (2020). This highlights that LP inoculation significantly restricted the growth of protein-degrading microbes and reserved the protein in intercropped silages. In addition, both CK-M and LP-M intercropped silages had significantly lower ammonia-N content compared to other intercropped ratios which might be related to the rapid acidification in these silage with greater production of LA and AA contents. In the current study, the protein content increased with the increased seed-sowing ratio of Lablab in intercropping, and LP silages had greater CP content compared to CK silages. A study has reported that LP treatment increased the contents of CP in mixed silages of amaranth and rice straw (Mu et al., 2020), which may be caused by the regulation of the bacterial community *via* LP inoculant in the intercropped silage, especially the proportion of *Lactobacillus*, which relates to the nutritional quality. Moreover, the increased contents of CP within the same group (LP or CK) were directly related to the seed-sowing ratio of Lablab as it is well-known that Lablab is a protein-rich crop (Hailin et al., 2017).

The dry matter content is an important index of the nutritional preservation of forages (Hu et al., 2009). We found that the DM of CK-L silage was greater than that of other silages after 60 d of ensiling, highlighting that neither Lablab mixing ratio nor LP inoculation influenced the DM content after ensiling. The contents of structural carbohydrates such as NDF and ADF were lower in the LP-H silage compared to other treatments. Similarly, a previous study reported that LP inoculation reduced the NDF and ADF contents in *Pennisetum sinese* silage (Li et al., 2021). This could be exemplified by the regulation of microbial community *via* LP inoculant which hydrolyzed the more digestible cell wall fractions by fibrinolytic enzyme and acids production during silage fermentation (Yan et al., 2019). Lignin is considered to be an indigestible carbohydrate in the process of animal digestion or ensiling (Desta et al., 2016); however, the lowest ADL was detected in both LP-H and CK-H silages. This further demonstrated that there might exist a biological delignification effect between LP inoculation and intercropping. However, it was fascinating to observe that the intercropped silages having lower seed-sowing ratios of Lablab with LP inoculation had lower ADL content compared to CK. This highlights the biological delignification effect of LP inoculation which can increase the permeability of plant cell walls and thereby increase the accessibility of hydrolytic enzymes or acids to polysaccharides (Li et al., 2018). The suitable fiber content of silages is helpful to animal digestion and health (Mcdonald et al., 1991). As expected, the LP-H silage showed a greater RFV compared to other groups, indicating that inoculated LAB improved the nutritional quality of Lablab and sweet sorghum mixed silage in agreement with the conclusions of Ni et al. (2018). Moreover, the NDF and ADF contents decreased with an increased ratio of Lablab in mixed silages, highlighting that the sweet sorghum ratio was the key factor contributing to higher levels of NDF and ADF.

Ensiling is a complex process that is regulated by the microbial community (Xu et al., 2021). Based on the information of the relative abundance of bacteria at the genus level, *Lactobacillus* and *Weissella* were dominant genera in CK-L and CK-M, but *Lactobacillus* was the prevalent genus in the LP group. The *Lactobacillus* genus is known to dominate the ensiling process under an airtight environment and could grow vigorously during the later phase of fermentation due to its greater acid resistance (Li et al., 2021). Similarly, a previous study has established that *Lactobacillu*s was the most abundant genus in barley silage with or without LAB inoculants after 60 d of ensiling (Liu et al., 2019). However, the *Weissella* genus is usually regarded as an early colonizer (Dellaglio and Torriani, 1986) and succeeds by acid-resistant *Lactobacillus* because of pH decline as fermentation begins (Graf et al., 2016). Moreover, it was quite fascinating to know that the relative abundance of *Lactobacillus* gradually increased, while the relative abundance of *Weissella* gradually decreased with an increased seed-sowing ratio of Lablab in intercropping. Similarly, Zeng et al. (2020) reported that corn and soybean intercropped silages had a greater relative abundance of *Lactobacillus* compared to *Weissella* and concluded that intercropping provides beneficial fermentation effects due to the difference in nutritional compositions of intercropped crops. Moreover, the relative abundance of LP in the LP silages was higher than that of CK silages. This might be related to the synergistic effects of intercropping which provided excellent conditions for inoculated bacteria to dominate in the fermentation process in accordance with the report of Li et al. (2022).

The silage process is largely related to microbial communities and biochemical reactions, and the fermentation of silage is largely dependent on the microbial community and a series of end products (Zhao et al., 2021; Li et al., 2022). In the present study, the genus *Lactobacillus* had a positive correlation with CP and LA, while showing a negative correlation with pH. Similar interactions were found by Mcallister et al. (2018) who reported that *Lactobacillus* could produce LA to reduce pH and inhibit proteolysis. On the contrary, *Weissella* was positively correlated with AA and negatively correlated with CP suggesting that the presence of *Weissella* in silage is not suitable for quality fermentation. Moreover, the addition of LP could enhance the relative abundance of *Lactobacillus* which creates acidic conditions and suppress the growth of undesirable microbes, such as *Weissella*, ultimately leading to quality fermentation.

## Conclusion

The intercropping and addition of LP inoculation significantly increased the LA content and decreased the pH value, AA, and ammonia-N content in the mixed silages. The intercropping or LP inoculation or both showed a positive effect on the bacterial community. The *Lactobacillus* was the most prevalent genus in LP groups compared to the CK groups and was positively associated with the LA and CP, while negatively correlated with ammonia-N. The LP was the prevalent specie in LP silages compared to CK silages, and its relative abundance increased with an increased seed-sowing ratio of Lablab in intercropping. Overall, better fermentation quality and preservation of nutritional components were achieved when the seed-sowing ratio of Lablab and sweet sorghum was ≥ 5:1, especially in LP-inoculated silages. Therefore, it is recommended to intercrop the Lablab and sweet sorghum at seed-sowing ratios of ≥ 5:1 for quality silage production in the saline-alkaline region.

## Data availability statement

The datasets presented in this study can be found in online repositories. The names of the repository/repositories and accession number(s) can be found at: https://www.ncbi.nlm.nih.gov/, PRJNA885981.

## Author contributions

JZho: funding acquisition, supervision, and conceptualization. HL: writing original draft and visualization. TW: investigation, methodology, and visualization. JZha and JS: data curation and supervision. ZL: supervision and project administration. FH, YL, and TX: resources and methodology. MT: reviewing. All authors contributed to the article and approved the submitted version.

## Funding

This research was funded by the Strategic Priority Research Program of the Chinese Academy of Sciences (XDA26040201) and the Key Deployment Project of the Chinese Academy of Sciences (KFZD-SW-113).

## Conflict of interest

The authors declare that the research was conducted in the absence of any commercial or financial relationships that could be construed as a potential conflict of interest.

## Publisher's note

All claims expressed in this article are solely those of the authors and do not necessarily represent those of their affiliated organizations, or those of the publisher, the editors and the reviewers. Any product that may be evaluated in this article, or claim that may be made by its manufacturer, is not guaranteed or endorsed by the publisher.

## Supplementary material

Supplementary Figure 1 The principal coordinates analysis (PCoA) of bacterial community dissimilarities of intercropped silages with or without LP. CK, sterile water; LP, *Lactobacillus plantarum*; L, Lablab and sweet sorghum seed-sowing ratio of 1:1; M, Lablab and sweet sorghum seed-sowing ratio of 5:1; and H, Lablab and sweet sorghum seed-sowing ratio of 9:1.

## References

[B1] AOAC and AOMA. (1990). Association of official analytical chemists. Official Methods Anal. 1, 69–90.

[B2] Arasu M. V. Jung M.-W. Kim D. H. Ilavenil S. Jane M. Park H. S.. (2014). Enhancing nutritional quality of silage by fermentation with *Lactobacillus plantarum*. Indian J. Microbiol. 54, 396–402. 10.1007/s12088-014-0473-9 25320437 PMC4186939

[B3] Broderick G. Kang J. (1980). Automated simultaneous determination of ammonia and total amino acids in ruminal fluid and *in vitro* media. J. Dairy Sci. 63, 64–75. 10.3168/jds.S0022-0302(80)82888-8 7372898

[B4] Chen L. Dong Z. Li J. Shao T. (2019). Ensiling characteristics, in vitro rumen fermentation, microbial communities and aerobic stability of low-dry matter silages produced with sweet sorghum and alfalfa mixtures. J. Sci. Food Agric. 99, 2140–2151. 10.1002/jsfa.9406 30298699

[B5] Dellaglio F. Torriani S. J. J. O. (1986). DNA-DNA homology, physiological characteristics and distribution of lactic acid bacteria isolated from maize silage. J. Appl. Bacteriol. 60, 83–92. 10.1111/j.1365-2672.1986.tb03363.x

[B6] Desta S. T. Yuan X. Li J. Shao T. (2016). Ensiling characteristics, structural and nonstructural carbohydrate composition and enzymatic digestibility of Napier grass ensiled with additives. Bioresour. Technol. 221, 447–454. 10.1016/j.biortech.2016.09.068 27668877

[B7] Dong M. Li Q. Xu F. Wang S. Chen J. Li W. (2020). Effects of microbial inoculants on the fermentation characteristics and microbial communities of sweet sorghum bagasse silage. Sci. Rep. 10, 1–9. 10.1038/s41598-020-57628-0 31964930 PMC6972861

[B8] Dunière L. Sindou J. Chaucheyras-Durand F. Chevallier I. Thévenot-Sergentet D. (2013). Silage processing and strategies to prevent persistence of undesirable microorganisms. Animal Feed Sci. Technol. 182, 1–15. 10.1016/j.anifeedsci.2013.04.006

[B9] EFSA Panel on Additives Products or Substances used in Animal Feed (FEEDAP) Rychen G. Aquilina G. Azimonti G. Bampidis V. Bastos M. D.. (2017). Safety and efficacy of *Lactobacillus plantarum* DSM 29024 as a silage additive for all animal species. EFSA J. 15, e04675. 10.2903/j.efsa.2017.4675 32625265 PMC7009910

[B10] Filya I. Sucu E. Karabulut A. (2006). The effect of *Lactobacillus buchneri* on the fermentation, aerobic stability and ruminal degradability of maize silage. J. Appl. Microbiol. 101, 1216–1223. 10.1111/j.1365-2672.2006.03038.x 17105551

[B11] Graf K. Ulrich A. Idler C. Klocke M. (2016). Bacterial community dynamics during ensiling of perennial ryegrass at two compaction levels monitored by terminal restriction fragment length polymorphism. J. Appl. Microbiol. 120, 1479–1491. 10.1111/jam.13114 26923533

[B12] Hailin L. Huifen D. Huaqin X. Ao S. (2017). Nutritional quality of mixed silage of *Lablab purpureus* and sweet sorghum. Anim. Husb. Feed Sci. 9, 398–416. 10.19578/j.enki.ahfs.2017.06.014

[B13] Hou X. Jia Y. Bao J. Zhang J. Fan M. Zhao M. (2018). Effect of soil saline-alkaline degree on growth period and agronomic characters of *Avena sativa* L. Animal Husbandry Feed Science 39, 45–49. Available online at: https://www.cabdirect.org/cabdirect/abstract/20183326800

[B14] Hu W. Schmidt R. J. Mcdonell E. E. Klingerman C. M. Kung L. (2009). The effect of *Lactobacillus buchneri* 40788 or *Lactobacillus plantarum* MTD-1 on the fermentation and aerobic stability of corn silages ensiled at two dry matter contents. J. Dairy Sci. 92, 3907–3914. 10.3168/jds.2008-1788 19620673

[B15] Khota W. Pholsen S. Higgs D. Cai Y. (2017). Fermentation quality and in vitro methane production of sorghum silage prepared with cellulase and lactic acid bacteria. Asian-Aust. J. Anim. Sci. 30, 1568. 10.5713/ajas.16.0502 28728399 PMC5666192

[B16] Li H. Zeng T. Du Z. Dong X. Xin Y. Wu Y.. (2022). Assessment on the fermentation quality and bacterial community of mixed silage of Faba bean with forage Wheat or Oat. Front. Microbiol. 13, 875819. 10.3389/fmicb.2022.875819 35602069 PMC9114351

[B17] Li J. Yuan X. Dong Z. Mugabe W. Shao T. (2018). The effects of fibrolytic enzymes, cellulolytic fungi and bacteria on the fermentation characteristics, structural carbohydrates degradation, and enzymatic conversion yields of *Pennisetum sinese* silage. Bioresour. Technol. 264, 123–130. 10.1016/j.biortech.2018.05.059 29800772

[B18] Li P. Lu Y. Zhao M. Chen L. Zhang C. Cheng Q.. (2021). Effects of phenyllactic acid, lactic acid bacteria, and their mixture on fermentation characteristics and microbial community composition of Timothy silage. Front. Microbiol. 12, 743433. 10.3389/fmicb.2021.743433 34975781 PMC8716789

[B19] Liu B. Huan H. Gu H. Xu N. Shen Q. Ding C. (2019). Dynamics of a microbial community during ensiling and upon aerobic exposure in lactic acid bacteria inoculation-treated and untreated barley silages. Bioresour. Technol. 273, 212–219. 10.1016/j.biortech.2018.10.041 30447622

[B20] Mcallister T. A. Dunière L. Drouin P. Xu S. Wang Y. Munns K.. (2018). Silage review: Using molecular approaches to define the microbial ecology of silage. J. Dairy Sci. 101, 4060–4074. 10.3168/jds.2017-13704 29685277

[B21] Mcdonald P. Henderson A. Heron S. J. E. (1991). The Biochemistry of Silage. Chalcombe: Chalcombe Publications.

[B22] Mu L. Xie Z. Hu L. Chen G. Zhang Z. (2020). Cellulase interacts with *Lactobacillus plantarum* to affect chemical composition, bacterial communities, and aerobic stability in mixed silage of high-moisture amaranth and rice straw. Bioresour. Technol. 315, 123772. 10.1016/j.biortech.2020.123772 32653750

[B23] Mudryj A. N. Yu N. Aukema H. M. (2014). Nutritional and health benefits of pulses. Appl. Physiol. Nutr. Metabol. 39, 1197–1204. 10.1139/apnm-2013-0557 25061763

[B24] Ni K. Zhao J. Zhu B. Su R. Pan Y. Ma J.. (2018). Assessing the fermentation quality and microbial community of the mixed silage of forage soybean with crop corn or sorghum. Bioresource Technol. 265, 563–567. 10.1016/j.biortech.2018.05.097 29861298

[B25] Oliveira A. S. Weinberg Z. G. Ogunade I. M. Cervantes A. a,.P Arriola K. G.. (2017). Meta-analysis of effects of inoculation with homofermentative and facultative heterofermentative lactic acid bacteria on silage fermentation, aerobic stability, and the performance of dairy cows. J. Dairy Sci. 100, 4587–4603. 10.3168/jds.2016-11815 28342607

[B26] Pahlow G. Muck R. E. Driehuis F. Elferink S. J. O. Spoelstra S. F. (2003). Microbiology of ensiling. Silage Sci. Technol. 42, 31–93. 10.2134/agronmonogr42.c2

[B27] Rebello C. J. Greenway F. L. Finley J. W. (2014). Whole grains and pulses: A comparison of the nutritional and health benefits. J. Agric. Food Chem. 62, 7029–7049. 10.1021/jf500932z 24992700

[B28] Ren H. Feng Y. Pei J. Li J. Wang Z. Fu S.. (2020). Effects of *Lactobacillus plantarum* additive and temperature on the ensiling quality and microbial community dynamics of cauliflower leaf silages. Bioresour. Technol. 307, 123238. 10.1016/j.biortech.2020.123238 32247271

[B29] Restelatto R. Novinski C. O. Pereira L. M. Silva E. P. Volpi D. Zopollatto M.. (2019). Chemical composition, fermentative losses, and microbial counts of total mixed ration silages inoculated with different *Lactobacillus* species. J. Anim. Sci. 97, 1634–1644. 10.1093/jas/skz030 30715358 PMC6447279

[B30] Su R. Ni K. Wang T. Yang X. Zhang J. Liu Y.. (2019). Effects of ferulic acid esterase-producing *Lactobacillus fermentum* and cellulase additives on the fermentation quality and microbial community of alfalfa silage. PeerJ 7, e7712. 10.7717/peerj.7712 31608168 PMC6788448

[B31] Sun J. Wang T. Huang F. Liu Y. Shi W. Ma C.. (2021). Silage fermentation: a potential microbial approach for the forage utilization of *Cyperus esculentus* L. by-product. Fermentation 7, 273. 10.3390/fermentation7040273

[B32] Van Soest P. J. Robertson J. B. Lewis B. A. (1991). Methods for dietary fiber, neutral detergent fiber, and nonstarch polysaccharides in relation to animal nutrition. J. Dairy Sci. 74, 3583–3597. 10.3168/jds.S0022-0302(91)78551-2 1660498

[B33] Wang T. Teng K. Cao Y. Shi W. Xuan Z. Zhou J.. (2020). Effects of *Lactobacillus hilgardii* 60TS-2, with or without homofermentative *Lactobacillus plantarum* B90, on the aerobic stability, fermentation quality and microbial community dynamics in sugarcane top silage. Bioresource Technol. 312, 123600. 10.1016/j.biortech.2020.123600 32531735

[B34] Xia T. Wang T. Sun J. Shi W. Liu Y. Huang F.. (2022). Modulation of fermentation quality and metabolome in co-ensiling of *Sesbania cannabina* and sweet sorghum by lactic acid bacterial inoculants. Front. Microbiol. 13, 851271–851271. 10.3389/fmicb.2022.851271 35401441 PMC8988063

[B35] Xu H. Sun L. Na N. Wang C. Yin G. Liu S.. (2021). Dynamics of bacterial community and fermentation quality in *Leymus chinensis* silage treated with lactic acid bacteria and/or water. Front. Microbiol. 12, 717120. 10.3389/fmicb.2021.717120 34803939 PMC8595406

[B36] Yan Y. Li X. Guan H. Huang L. Ma X. Peng Y.. (2019). Microbial community and fermentation characteristic of Italian ryegrass silage prepared with corn stover and lactic acid bacteria. Bioresource Technol. 279, 166–173. 10.1016/j.biortech.2019.01.107 30721817

[B37] Yang F. Wang Y. Zhao S. Wang Y. (2020). *Lactobacillus plantarum* inoculants delay spoilage of high moisture alfalfa silages by regulating bacterial community composition. Front. Microbiol. 11, 1989. 10.3389/fmicb.2020.01989 32903392 PMC7434841

[B38] Yang L. Yuan X. Li J. Dong Z. Shao T. (2019). Dynamics of microbial community and fermentation quality during ensiling of sterile and nonsterile alfalfa with or without *Lactobacillus plantarum* inoculant. Bioresour. Technol. 275, 280–287. 10.1016/j.biortech.2018.12.067 30594838

[B39] Yu J. (2015). Study on Mixed Silage Effect of Lablab and Several Grasses. Master, Hunan Agricultural University (In Chinese).

[B40] Zeng T. Li X. Guan H. Yang W. Liu W. Liu J.. (2020). Dynamic microbial diversity and fermentation quality of the mixed silage of corn and soybean grown in strip intercropping system. Bioresource Technol. 313, 123655. 10.1016/j.biortech.2020.123655 32559709

[B41] Zeng T. Wu Y. Xin Y. Chen C. Du Z. Li X.. (2022). Silage quality and output of different maize–soybean strip intercropping patterns. Fermentation 8, 174. 10.3390/fermentation8040174

[B42] Zhang S. J. Chaudhry A. S. Osman A. Shi C. Q. Edwards G. R. Dewhurst R. J.. (2015). Associative effects of ensiling mixtures of sweet sorghum and alfalfa on nutritive value, fermentation and methane characteristics. Anim. Feed Sci. Technol. 206, 29–38. 10.1016/j.anifeedsci.2015.05.00630298699

[B43] Zhang X. Ke W. Ding Z. Xu D. Wang M. Chen M.. (2022). Microbial mechanisms of using feruloyl esterase-producing *Lactobacillus plantarum* A1 and grape pomace to improve fermentation quality and mitigate ruminal methane emission of ensiled alfalfa for cleaner animal production. J. Environ. Manage. 308, 114637. 10.1016/j.jenvman.2022.114637 35124318

[B44] Zhao S. Wang Y. Yang F. Wang Y. Zhang H. (2020). Screening a *Lactobacillus plantarum* strain for good adaption in alfalfa ensiling and demonstrating its improvement of alfalfa silage quality. J. Appl. Microbiol. 129, 233–242. 10.1111/jam.14604 32027450

[B45] Zhao S. Yang F. Wang Y. Fan X. Feng C. Wang Y. (2021). Dynamics of fermentation parameters and bacterial community in high-moisture alfalfa silage with or without lactic acid bacteria. Microorganisms 9, 1225. 10.3390/microorganisms9061225 34200084 PMC8226466

