# Supplementary figures and images for: Influence of Lactobacillus plantarum inoculation on the silage quality of intercropped Lablab purpureus and sweet sorghum grown in saline-alkaline region
Source: Front Microbiol. 2022 Dec 2;13:1059551. doi: 10.3389/fmicb.2022.1059551 (PMC9755603; doi:10.3389/fmicb.2022.1059551)

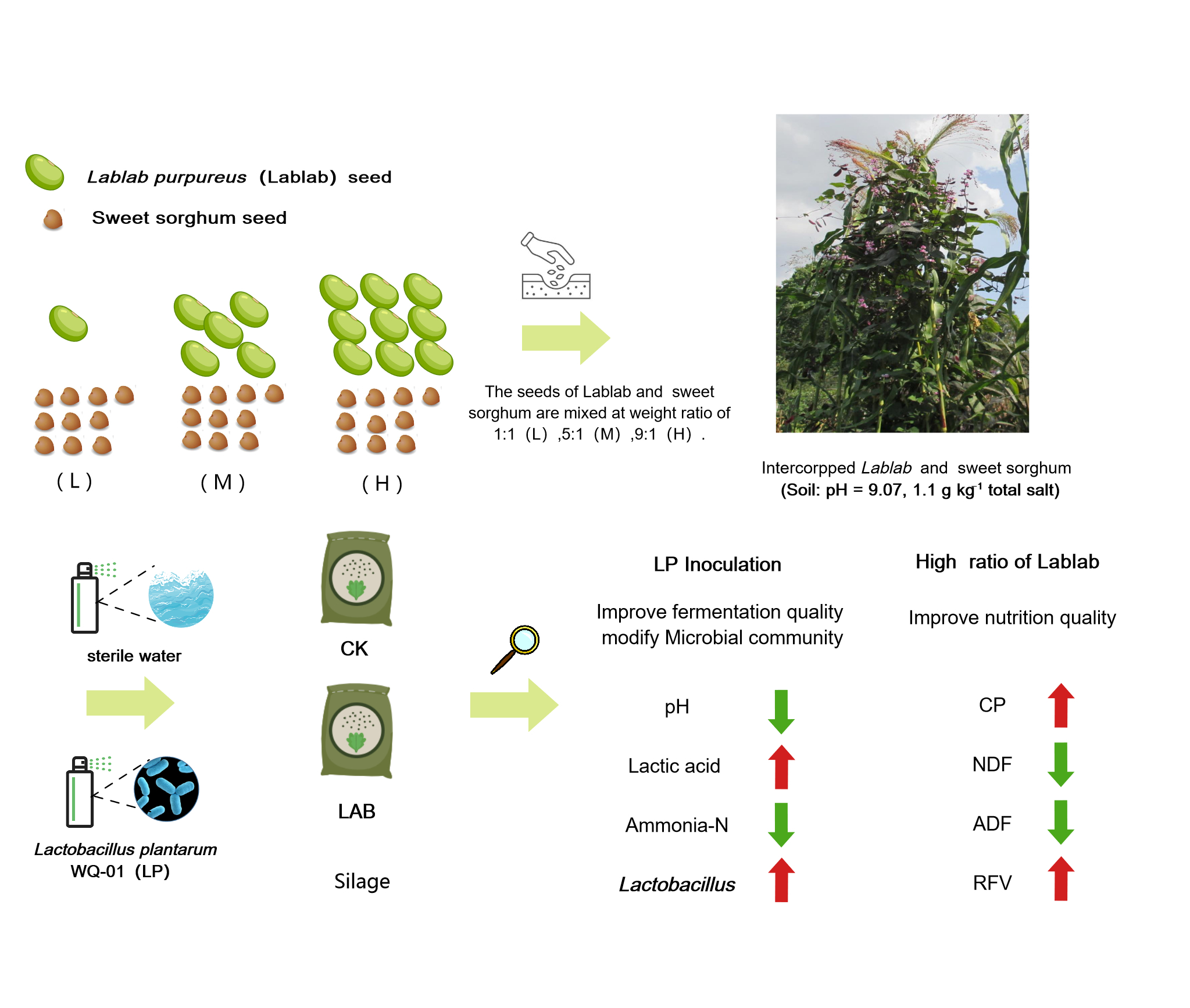

Supplement: Supplementary Figure 1 — The principal coordinates analysis (PCoA) of bacterial community dissimilarities of intercropped silages with or without LP. CK, sterile water; LP, Lactobacillus plantarum; L, Lablab and sweet sorghum seed-sowing ratio of 1:1; M, Lablab and sweet sorghum seed-sowing ratio of 5:1; and H, Lablab and sweet sorghum seed-sowing ratio of 9:1. [file Image_1.JPEG]
