# Supplementary material for: Influence of Lactobacillus plantarum inoculation on the silage quality of intercropped Lablab purpureus and sweet sorghum grown in saline-alkaline region
Source: Front Microbiol. 2022 Dec 2;13:1059551. doi: 10.3389/fmicb.2022.1059551 (PMC9755603; doi:10.3389/fmicb.2022.1059551)
Supplement: Supplementary file 2 [file Data_Sheet_1.docx]

**Table S1.**

| Table S1. Alpha diversity of bacterial of Lablab and sweet sorghum intercropped silages. | | | | | | |
| --- | --- | --- | --- | --- | --- | --- |
| Alpha diversity indices | CK | | | LP | | |
|  | L | M | H | L | M | H |
| Observed species | 156.17 | 140.17 | 167.33 | 181.83 | 144 | 177.17 |
| Shannon | 2.32 | 1.64 | 1.52 | 0.57 | 0.38 | 0.38 |
| Simpson | 0.66 | 0.54 | 0.32 | 0.11 | 0.07 | 0.06 |
| Ace | 211.07 | 195.8 | 220.28 | 246.94 | 200.32 | 235.82 |
| Chao1 | 227.42 | 210.33 | 262.09 | 274.02 | 220.57 | 251.52 |
| Good’s coverage | 0.999 | 0.999 | 0.999 | 0.998 | 0.999 | 0.999 |
| CK, Sterile water; LP, *Lactobacillus plantarum*; L, Lablab and sweet sorghum sowing weight ratio of 1:1; M, Lablab and sweet sorghum sowing weight ratio of 5:1; H, Lablab and sweet sorghum sowing weight ratio of 9:1. | | | | | | |
